# Photocatalytic Degradation of Rhodamine B Using 1D CuO/TiO_2_ Nanofibers Synthesized via the Electrospinning Method

**DOI:** 10.3390/ma18184252

**Published:** 2025-09-11

**Authors:** Shouzhen Duan, Wanjun Zhang, Xiaoyan Wang, Youqing Zhao, Hui Nan, Guijun Yang

**Affiliations:** 1 Chemical Engineering Institute, Qinghai University, Xining 810016, China; 2 New Energy Branch, Huanghe Hydropower Development Co., Ltd., Xining 810006, China

**Keywords:** TiO_2_, CuO, electrospinning method, PN junction, photocatalytic

## Abstract

This research was designed to improve the separation efficiency of photogenerated carriers in TiO_2_ through the construction of a PN heterojunction. The motivation behind this was to tackle the problems of the narrow light response range and the high electron-hole recombination rate of TiO_2_. By simple one-step implementing electrospinning and calcination procedures, CuO/TiO_2_ PN heterojunction nanofibers were successfully synthesized. XRD and SEM analyses confirm that the heterojunction is a nanofiber structure composed of TiO_2_ and CuO, with the TiO_2_ containing anatase and rutile phases. The PL reveals that the fluorescence intensity of the heterojunction is lower compared to that of pure TiO_2_, and this implies a remarkable enhancement in the carrier separation efficiency. Under xenon light irradiation, for the optimized sample, the degradation rate of RhB exceeds 80%. This degradation rate is 68% higher than that of pure TiO_2_. The improvement in photocatalytic performance can be ascribed to the efficient charge separation driven by the built-in electric field within the PN junction and the extended light absorption range. The photoelectrochemical test further verified that the photocurrent density of the heterojunction system was 52.42% higher than that of the single TiO_2_, providing a new strategy for designing efficient photocatalytic systems.

## 1. Introduction

In the 21st century, the governance of environmental pollution and the pursuit of sustainable development have emerged as significant challenges confronting the global community [1,2]. In the field of industrial production, a large amount of organic dye wastewater such as Rhodamine B (RhB), methyl orange and methylene blue generated by industries such as printing and dyeing, textile, leather, and papermaking causes severe pollution to water environment, soil and atmosphere [3]. Among them, the typical pollutant RhB is difficult to be completely degraded by traditional treatment technologies due to its high chemical stability and strong carcinogenicity, leading to the pollutants entering soil, atmosphere and biological chain through water cycle and forming cross-media complex pollution [4]. Photocatalytic technology has emerged as a prominent approach in the field of environmental science, particularly in the context of organic pollutant treatment. Its distinct advantages, including environmental benignity, complete degradation of pollutants, and efficient utilization of solar energy [5,6], have positioned it as a significant breakthrough in addressing the challenges associated with organic pollution. This technology not only offers a sustainable solution but also holds great promise for the development of more efficient and eco-friendly treatment methods in the future. Semiconductor photocatalytic materials, such as TiO_2_, have demonstrated significant potential in the realm of environmental purification [7,8]. This is attributed to their favorable attributes, including stable chemical properties, non-toxicity, and low cost [9]. However, the TiO_2_ single material has some inherent limitations. For instance, its light response range is narrow, being confined to the ultraviolet region [10]. Additionally, it has a high recombination rate of photogenerated carriers and a relatively limited specific surface area. These issues seriously impede the efficiency of its practical applications. In order to break through these technical bottlenecks, TiO_2_ has been modified and optimized by semiconductor recombination [11] and metal doping [12] to significantly expand its visible light absorption capacity and improve its quantum efficiency, which has made significant progress in the field of organic dye degradation.

CuO is a typical p-type semiconductor with excellent chemical stability [13]. TiO_2_ is a typical n-type semiconductor, and the two can be combined in various ways to form a PN junction [14]. The PN junction is a space charge region formed by the contact between p-type semiconductors and n-type semiconductors. Its unique energy band structure and charge separation mechanism show key advantages in the field of photocatalysis [15,16], such as efficient charge separation and inhibition recombination, broadening the photoresponse range, improving the interfacial reaction activity, optimizing the stability and recyclability, and extending the versatility.

There are various methods for preparing composite materials, such as chemical deposition [17] and solvothermal reaction [18], but most of them involve high-temperature and high-pressure reaction conditions and have limited yields. Electrospinning technology has the advantages of being able to prepare nanofibers, controllable process parameters and other conditions, a wide range of material adaptation, low cost and easy to upgrade, controllable structural parameters, and environmentally friendly potential. These advantages enable electrospinning to be applied to the fabrication of photocatalytic materials [19]. Due to the limitation of the hydrolysis of titanium precursor, researchers often use the way of preparing TiO_2_ fiber first and attaching copper oxide to it [20]. This composite method prevents effective contact and efficient electron transfer between CuO and TiO_2_.

In conclusion, the formation of a PN junction by CuO and TiO_2_ can alter their photocatalytic performances. Therefore, in this experiment, the one-dimensional CuO/TiO_2_ nanofibers were prepared by electrospinning combined with annealing process to study their performance in photocatalytic degradation of RhB. Through electrochemical and UV-Vis related characterization, it was determined that the PN junction formed by CuO and TiO_2_ plays an important role in accelerating the separation and transmission of charge carriers.

## 2. Materials and Methods

### 2.1. Materials

In this study, we used several chemical reagents, including copper acetate (Cu (OAc)_2_ A.R.) purchased from Tianjin Baishi Chemical Co., Ltd. (Tianjin, China), tetrabutyl titanate (TBOT A.R.) and isopropanol (A.R.) purchased from Shanghai Aladdin Biochemical Technology Co., Ltd. (Shanghai, China), glacial acetic acid (AcOH A.R.) and sodium hydroxide (A.R.) purchased from Sichuan Xiling Science Co., Ltd. (Chengdu, China), polyvinylpyrrolidone (PVP A.R.), anhydrous sodium sulfate (A.R.) purchased from Sinopharm Chemical Reagent Co., Ltd. (Shanghai, China), ethanol (EtOH A.R.) purchased from Chengdu Kelong Chemicals Co., Ltd. (Chengdu, China), p-benzoquinone (A.R.) purchased from Shanghai McLyn Biochemical Technology Co., Ltd. (Shanghai, China), and Rhodamine B (RhB A.R.) purchased from Tianjin Kaitong Chemical Reagent Co., Ltd. (Tianjin, China). All the chemical reagents were used directly without further purification.

### 2.2. Sample Preparation

**Figure 1:**
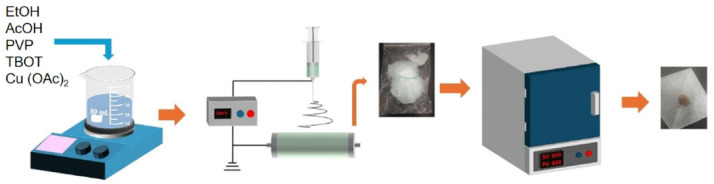
Schematic diagram of CuO/TiO_2_ preparation process.

In total, 4 g polyvinylpyrrolidone was dissolved into 10 mL absolute ethanol, and 2 mL glacial acetic acid was added to the stir well before adding 2 mL tetrabutyl titanate as the titanium source. Among them, absolute ethanol was the solvent, glacial acetic acid was the inhibitor, and polyvinylpyrrolidone was the template agent. The electrospinning solution was obtained after mixing and stirring for 10 h and standing for 2 h. The electrospinning solution was added into a 5 mL syringe, and the horizontal distance between the needle and the receiver was 15 cm. The parameters were set as follows: positive and negative high pressures were −2.5 kV and 18.0 kV, respectively, the injection rate of the injector was 0.6 mm/min, and the speed of the cylinder receiver was 100 r/min. The samples obtained by electrospinning were annealed in a muffle furnace (Kejing Material Technology Co., Ltd., Hefei, China)under the following conditions: heating rate of 1 °C/min, annealing temperature of 650 °C, and holding time of 2 h. After the temperature of the muffle furnace dropped to room temperature, the samples were removed, that is, the TiO_2_ nanofibers were obtained. Figure 1 is the schematic diagram of sample preparation.

The titanium source was converted to copper acetate as described above to obtain CuO. Based on the preparation of TiO_2_, different proportions of copper sources were added. The molar ratios of Ti and Cu sources were set at 10:1, 10:2, 10:3 and 10:4 to obtain the CuO/TiO_2_ composite samples of CR-1, CT-2, CT-3 and CT-4, respectively.

### 2.3. Characterization

The samples’ crystallinity was confirmed by XRD (D8 Bruker diffractometer, Bruker AXS, Karlsruhe, Germany). SEM (JSM-7900F, JEOL Ltd., Tokyo, Japan) was used to analyze the materials in order to ascertain their structure and form. X-ray photoelectron spectroscopy was used to evaluate the chemical states of the sample elements (SCIENTIFIC ESCALABXi+ XPS, Thermo Fisher Scientific Inc., Waltham, MA, USA). Diffuse reflectance spectroscopy (DRS) of the samples was assessed using a UV-Vis spectrophotometer (Cary 600, Agilet Technologies, Santa Clara, CA, USA). A three-electrode electrochemical analyzer and an electrochemistry workstation (CHI 660E, CH Instruments, Shanghai, China) were utilized to measure photoelectrochemical characteristics. The photoluminescence spectrum (PL) was acquired using a F-7000 fluorescence spectrophotometer (Hitachi High-Tech Corporation, Tokyo, Japan).

### 2.4. Photoelectrochemical Test

The 5 mg sample was dispersed using one milliliter of anhydrous ethanol (a), and the Nafion (20 μL) was spread using one milliliter of anhydrous ethanol (b). After that, each was put on a sensitive glass surface measuring 1 × 1 cm. The first one, (a), was coated five times with 10 μL and allowed to dry. In the second step, 20 μL of (b) was covered and allowed to dry. The electrolyte in the electrochemical test process was 0.5 mol/L Na_2_SO_4_.

### 2.5. Photocatalytic Experiment

To evaluate the photocatalytic activity of CuO/TiO_2_, RhB degradation was observed under a 300 W xenon light. Then, 20 mg of catalyst powder and 100 mL of RhB aqueous solution (10 mg/L) were added to a 150 mL glass for the experiments that followed.

After 40 min of darkness, the equilibrium between adsorption and desorption is reached. Next, periodically take 4 mL of the dye solution. The top layer of the cleaned solution should be removed once the samples have been centrifuged. UV-visible spectrophotometer (Cary 60, Agilent Technologies, Santa Clara, CA, USA) used to quantify absorption, then analyze and assess the data.

### 2.6. Free Radical Capture Experiments

The active substances that play a major role in the photocatalytic degradation process can be determined through free radical trapping experiments. Methanol (MeOH) was employed to trap holes, while benzoquinone (TBQ) and isopropanol (IPA) were captured superoxide free radicals (·O_2_^−^) and hydroxyl free radicals (·OH).

## 3. Results

### 3.1. Microstructural Analusis

**Figure 2:**
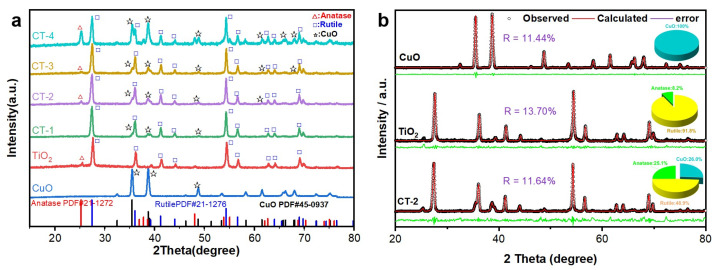
The XRD patterns of TiO_2_, CuO, CT-1, CT-2, CT-3 and CT-4 (**a**); the Rietveld analysis of CuO, TiO2 and CT-2 (**b**).

The phase composition of the sample was analyzed by XRD, as shown in Figure 2. The diffraction peak shape of each sample was clear, indicating that the photocatalyst crystallized well. It can be found that the characteristic peak of TiO_2_ at 25.30° shows the characteristic diffraction peak matching with the standard peak of anatase (PDF#21-1272), which belongs to the (101) and crystal plane of anatase type TiO_2_. The sharp peak shape and narrow FWHM of the peak confirm that the anatase phase in the material has a highly ordered crystal structure [21]. In addition, the characteristic diffraction peaks of rutile phase were observed at 27.45°, 36.09°, 41.23°, 54.32° and 56.64°in TiO_2_, and these peaks were assigned to (110), (101), (111), (211), (220) and (215) crystal planes of rutile TiO_2_, respectively. This indicates that both anatase and rutile phase structures exist in TiO_2_ of all samples. The characteristic peaks of CuO appeared at 36.45°, 42.30° and 50.43°, which were assigned to the (111), (200) and (220) crystal planes of CuO, respectively. The CuO/TiO_2_ composite material has been successfully synthesized.

It is worth noting that the intensity of the anatase main peak (101) shows a regular difference in the composite as the CuO content increases, indicating that the introduction of CuO may affect the crystal phase stability of TiO_2_. XRD results show that the CuO and TiO_2_ composite material is successfully prepared [22]. The XRD of CuO, TiO_2_ and CT-2 was refined, as shown in Figure 2b. The results indicated that in CT-2, CuO accounted for 26%, anatase accounted for 25.1%, and rutile phase accounted for 48.9%, all of which were expressed as mass ratios.

**Figure 3:**
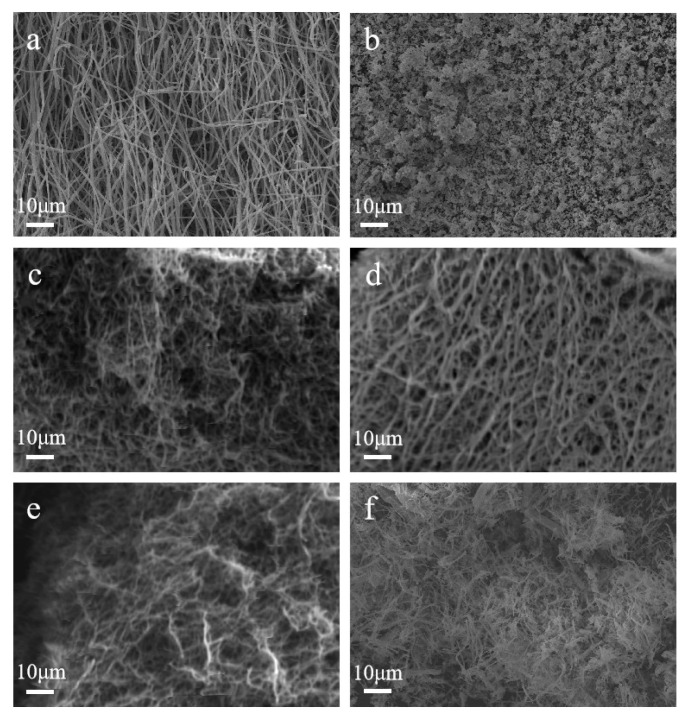
Electron microscope scans of different samples: (**a**) TiO_2_, (**b**) CuO, (**c**) CT-1, (**d**) CT-2, (**e**) CT-3, and (**f**) CT-4.

To further investigate the morphology of the material, the samples were examined by scanning electron microscopy. Figure 3a shows the microscopic morphology of pure TiO_2_, which can be observed to have regular one-dimensional nanofibers, while the microscopic morphology of pure CuO is irregular and granular. The microstructure of the composite samples showed the shape of one-dimensional nanofibers, and the nanofibers of CT-2 were relatively regular. The fibers of CT-3 began to cross-link. With the increase in CuO content, the shape of the nanofibers was destroyed, and CT-4 showed a fractured fiber. This indicates that the main phase of the composite sample is still TiO_2_, and the addition of CuO affects the microstructure change in the composite sample during calcination. In addition, the element distribution in CT-2 is observed by Figure S1, in which it can be seen that Ti, O and Cu elements are uniformly distributed in the composite sample CT-2.

**Figure 4:**
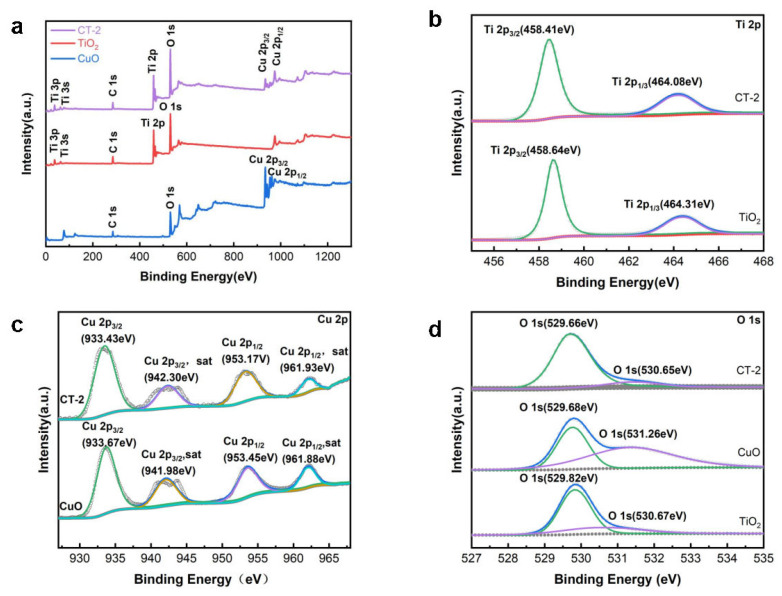
XPS survey (**a**), XPS spectra of Ti 2p (**b**), Cu 2p (**c**), and O 1s (**d**) of CuO, TiO_2_ and CuO/TiO_2_ composites were investigated.

The composition and valence states of elements in the samples were investigated by the XPS analysis method. As shown in Figure 4a, the XPS spectrum confirms that the composite material is composed of oxygen (O), titanium (Ti), and copper (Cu). In comparison with pure TiO_2_, the full spectrum of CT-2 exhibits additional 2p orbital characteristic peaks of Cu^2+^. Conversely, when compared to pure CuO, CT-2 shows additional 2p orbital characteristic peaks of Ti^4+^. These findings firmly confirm the successful synthesis of the CuO/TiO_2_ composite. Figure 4b presents the high resolution spectrum of Ti 2p. In the spectrum, two orbital peaks of the Ti element can be observed at approximately 458 eV and 464 eV. These peaks correspond to the 2p_3/2_ and 2p_1/2_ orbitals of the Ti element [23]. Notably, within the sample depicted in the figure, no additional peaks are detected. This absence of other peaks strongly suggests that the Ti element in this sample exists solely in the form of Ti^4+^ [24]. The binding energies of the Ti 2p_3/2_ and Ti 2p_1/2_ peaks in TiO_2_ are precisely located at 458.64 eV and 464.31 eV, respectively. In the case of CT-2, the Ti 2p_3/2_ and Ti 2p_1/2_ peaks are found at 458.41 eV and 464.08 eV, respectively. Compared to pure TiO_2_, a deviation in binding energies of 0.23~0.24 eV is evident, indicating that there is a certain electron transfer between TiO_2_ and CuO in CT-2.

The fine structure spectrum of the 2p orbital of Cu^2+^ is presented in Figure 4c. For the Cu element, four orbital peaks are detected at around 933 eV, 941.9 eV, 953 eV, and 961.9 eV. These peaks, respectively, correspond to the 2p_3/2_, 2p_3/2_ satellite peaks, 2p_1/2_, and 2p_1/2_ satellite peaks of the Cu^2+^ element, as reported in reference [25]. In the figure, no additional peaks are discerned in the sample. This observation strongly suggests that all the Cu elements within this sample are present in the form of Cu^2+^. Moreover, compared to pure CuO, the fine—structure peak of Cu in CT-2 exhibits a shift ranging from 0.24 eV to 0.28 eV. Figure 4d shows the O1s fine spectrum. The binding energies from high to low correspond to surface adsorb oxygen and lattice oxygen. The binding energy positions of lattice oxygen and oxygen vacancies in CT-2 are located between those of pure TiO_2_.

### 3.2. Catalytic Performane

**Figure 5:**
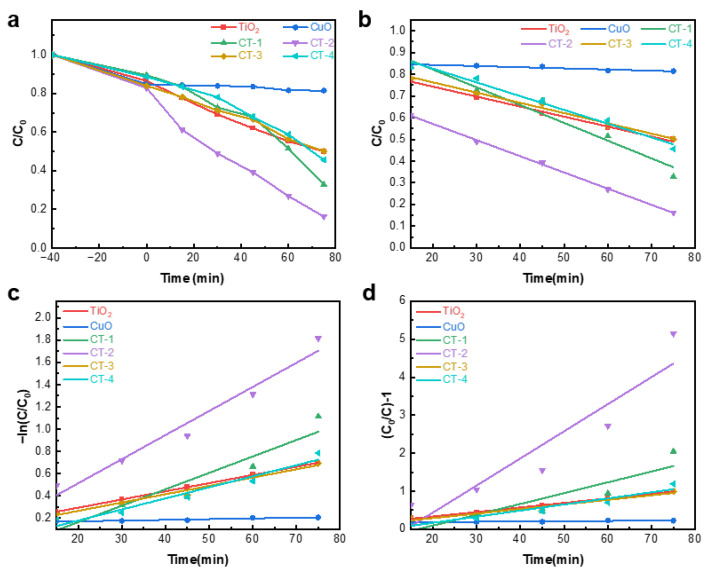
Degradation of rhodamine B plots for different specimens: (**a**) zero-level kinetic fit; (**b**) first-level kinetic fit; (**c**) second-level kinetic fit (**d**).

The degradation curve of RhB by the catalyst is shown in Figure 5a. The first 40 min in the figure represent the adsorption stage of RhB onto the catalyst, and then the light reaction begins. As can be seen from the Figure 5a, under the same conditions, CT-2 has the highest degradation efficiency for RhB and the CuO has almost no photodegradation effect on RhB. Figure 5b–d presents the zero-order, first-order and second-order kinetic fittings of the catalyst for RhB degradation. Table S1 shows the slopes and determination coefficients of these three kinetic fittings.

From Table S1, it can be seen that except for CuO, which has almost no photodegradation effect, the fitting results of the other samples all indicate that zero-order kinetics fitting has the highest goodness of fit, suggesting that this photodegradation reaction is most likely a pseudo-zero-order kinetics reaction. Among these three kinetic fittings, the slope of the composite sample CT-2 is the largest. Although the slope of the zero-order reaction kinetics CT-2 is not the largest, the difference from the largest CT-1 is not significant, and the slopes of the first-order and second-order kinetic fittings of CT-2 are multiples of the other samples. Therefore, the composite sample CT-2 has the strongest photodegradation ability, and thus CT-2 is the best-performing heterojunction constructed.

**Figure 6:**
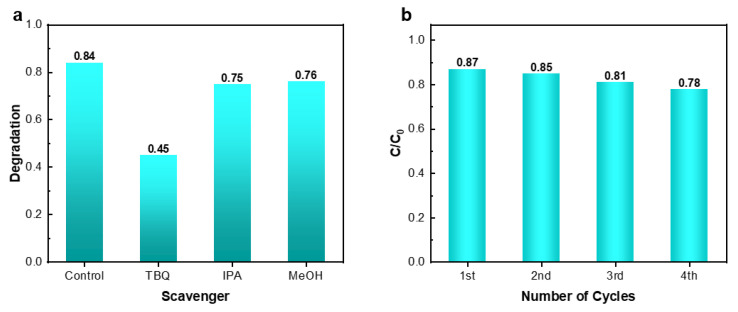
Graph of free radical trapping experiments (**a**) and Cyclic stability (**b**).

Figure 6a shows the capture experiments using p-benzoquinone (TBQ), isopropanol (IPA), and methanol (MeOH) as the capture agents. Here, TBQ captures superoxide anion (·O_2_^−^), IPA captures hydroxyl radical (·OH), and MeOH captures hole (h^+^). The experimental results show that after adding IPA and MeOH, the photocatalytic degradation efficiency of RhB by CT-2 slightly decreased. However, after adding TBQ, the degradation efficiency significantly decreased. This indicates that ·OH and h^+^ play a role in assisting the degradation process, while ·O_2_^−^ plays the main role in the photocatalytic degradation of this sample. To prove the good stability of the photocatalyst, as shown in Figure 6b, after 4 cycles, the degradation efficiency of RhB remained 89.7% of the first degradation.

### 3.3. Electrochmical Measurments

**Figure 7:**
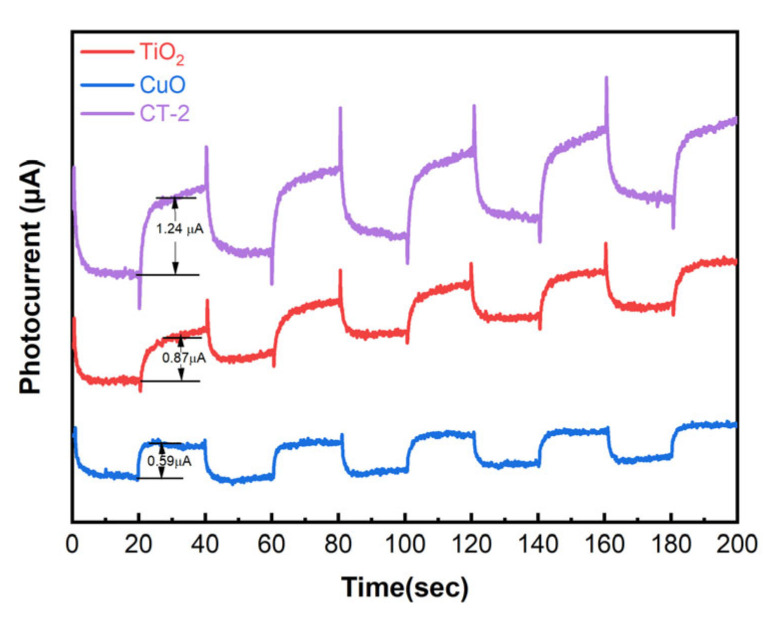
Optical current density plots of TiO_2_, CuO and CT-2.

In order to further investigate the transfer of photogenerated carriers in the samples, as shown in Figure 7, the curve relationship between the photocurrent density and time of TiO_2_, the control sample CuO, and the optimal composite sample CT-2 is presented. CT-2 has significantly higher photocurrent density than TiO_2_ and CuO.

**Figure 8:**
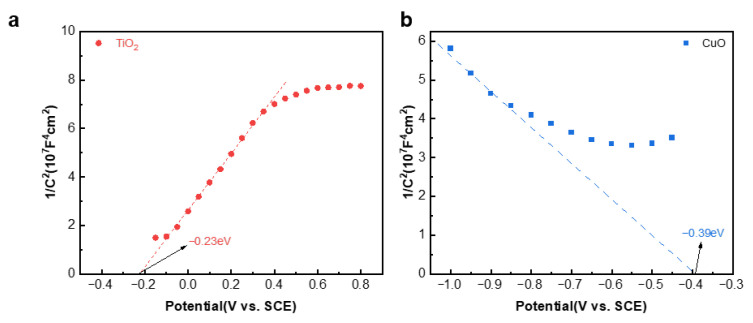
Mott–Schottky (M-S) diagrams of TiO_2_ (**a**), and CuO (**b**).

In order to investigate the possible heterojunction types in the composite materials, we conducted Mott–Schottky tests on TiO_2_ and CuO. As shown in Figure 8, since the slope of the M-S curve of TiO_2_ is positive, while that of CuO is negative, it indicates that TiO_2_ is an n-type semiconductor and CuO is a p-type semiconductor [26,27,28]. Using a saturated calomel electrode (SCE) as the reference electrode, the flat band potential of TiO_2_ is −0.23 V, and that of CuO is −0.39 V. After the conversion according to the Nernst equation Evs._SCE_ = Evs._NHE_ − 0.244 V [29], the flat band potentials of TiO_2_ and CuO under the standard hydrogen electrode are 0.01 V and −0.15 V, respectively. The flat band potential of an n-type semiconductor is higher than the conduction band potential by 0.1~0.3 V, while that of a p-type semiconductor is lower than the conduction band potential by 0.1~0.3 V. According to the literature, this is calculated as 0.2 V [30], resulting in the conduction band position of TiO_2_ being −0.19 V. The valence band potential of CuO is 0.05 V.

### 3.4. Optical and Electronic Properties

**Figure 9:**
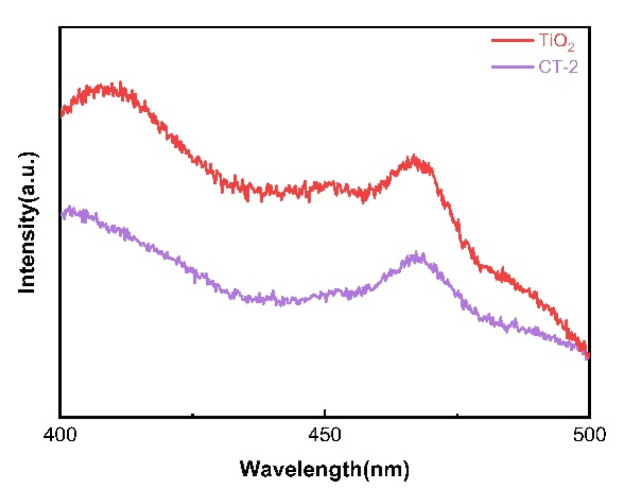
Photoluminescence spectra of TiO_2_ and CT-2.

Figure 9 shows the PL of the sample. The PL can be used for qualitative analysis of the photogenerated carrier separation efficiency of the photocatalyst. The better the separation and recombination efficiency of photogenerated carriers, the higher the photoluminescence intensity of the PL, and vice versa [16]. Among them, the luminescence intensity of the composite material CT-2 is significantly lower than that of TiO_2_. This indicates that the CT-2 has a higher photogenerated carrier separation efficiency.

**Figure 10:**
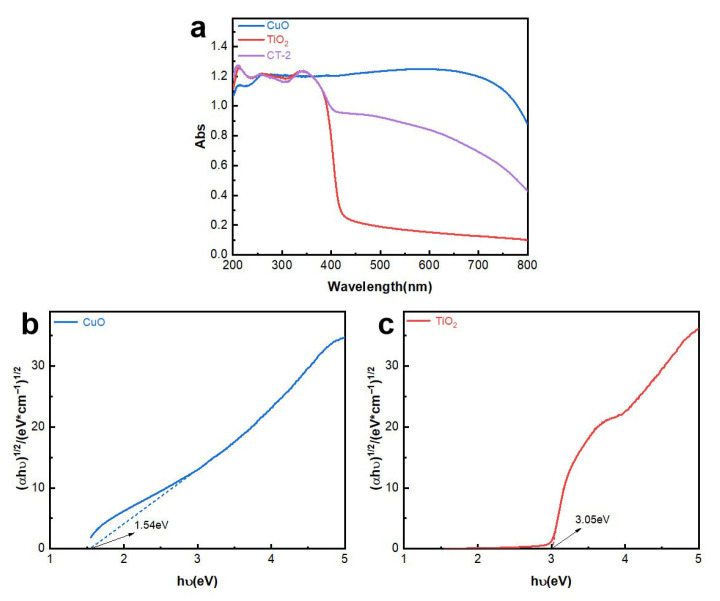
UV-Vis total spectrum of the sample: (**a**) band gap diagram of the sample; (**b**) CuO; (**c**) TiO_2_.

The UV-Vis diffuse reflectance spectrum of the catalyst is shown in Figure 10a. It can be seen that CuO has a strong absorption throughout the ultraviolet-visible wavelength range. The absorption band of TiO_2_ is around 400 nanometers, but the composite sample CT-2 has a significant absorption in the visible light region after adding CuO, successfully broadening the absorption range and extending the original ultraviolet absorption range to the visible light region. The band gap width of the photocatalyst was calculated according to the Kubelka-Munk formula [31], as shown in Figure 10b,c. The band gaps of CuO and TiO_2_ are 1.54 eV and 3.05 eV, respectively. Combining the band gaps of CuO and TiO_2_ and the formula Eg = ECB − EVB, the energy band structure of CuO and TiO_2_ can be determined, the specific data related to the structure can be seen in Table S2.

### 3.5. Photocatalytc Performance Mechanism

**Figure 11:**
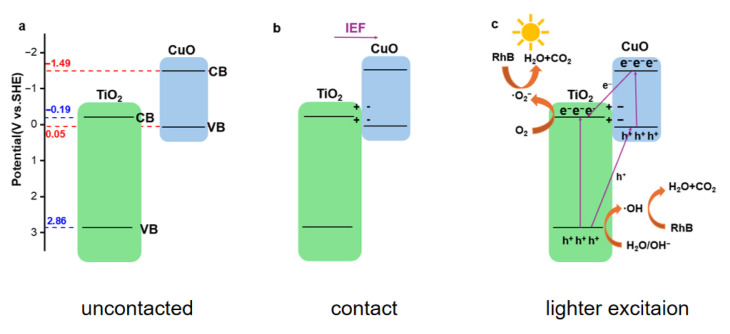
Energy level diagrams of uncontacted comparison samples (**a**); energy level diagrams of samples in contact (**b**); mechanism diagrams in light (**c**).

Based on the above research results, the electron transfer situation between TiO_2_ and CuO was analyzed, as shown in Figure 11. The conduction band position of TiO_2_ is −0.19 eV, and the valence band position is 2.86 eV; the conduction band position of CuO is −1.49 eV, and the valence band position is 0.05 eV. Among them, TiO_2_ is a typical n-type semiconductor, and CuO is a typical p-type semiconductor. When the two semiconductors form a PN junction, the electrons at the interface of TiO_2_ transfer to CuO, and the holes at CuO transfer to TiO_2_ [32]. It should be noted that the TiO_2_ mentioned here is actually a mixed phase consisting of rutile and anatase, due to their similar band structures with a difference of 0.2 eV. We believe that this difference has a relatively minor impact on the CuO/TiO_2_ PN heterojunction. Due to the local carrier transfer, an intrinsic electric field pointing from the TiO_2_ side to the CuO side is formed at the interface, as shown in Figure 11b. When the catalyst is excited under light conditions, the electrons at the valence band absorb photons and jump to the conduction band position. Because of the intrinsic electric field, the excess electrons on the CuO conduction band will transfer to the conduction band position of TiO_2_, and the excess holes on the TiO_2_ valence band will transfer to the valence band position of CuO. At the conduction band position of TiO_2_, some dissolved oxygen in the solution captures electrons and forms superoxide anion, which further plays a role in photocatalysis. In the experiment of photocatalytic degradation of RhB, superoxide anion decomposes RhB molecules into H_2_O and CO_2_, which is the main mechanism of the degradation experiment, as shown in Figure 11c. Additionally, some H_2_O or OH^-^ capture holes at the valence band position of CuO to form hydroxyl radicals ·OH.

## 4. Conclusions

In this study, TiO_2_/CuO PN heterojunction one-dimensional nanofiber materials were successfully fabricated through electrospinning combined with high-temperature calcination technology. The band matching at the heterojunction interface and the built-in electric field effect significantly optimized the separation efficiency of photogenerated carriers. This was verified by the decrease in PL spectral intensity and the photocurrent response. Under xenon light irradiation, the degradation efficiency of CT-2 for RhB (10 mg/L) reached over 80%, which is much higher than that of pure TiO_2_. The improvement in performance is mainly attributed to the efficient charge separation and transfer driven by the p-n junction. This work provides a theoretical basis for the design of p-n junction photocatalysts based on band engineering.

## Data Availability

The original contributions presented in this study are included in the article/Supplementary Materials. Further inquiries can be directed to the corresponding author.

## Supplementary Materials

The following supporting information can be downloaded at https://www.mdpi.com/article/10.3390/ma18184252/s1, Figure S1: EDS image of CuO/TiO_2_; Table S1: Slope (K value) and coefficient of determination (R^2^) in various fitted plots; Table S2: Energy level data statistics; Table S3: Efficiency comparison table of CuO/TiO_2_ materials [33,34,35,36,37].
