# Supplementary material for: Photocatalytic Degradation of Rhodamine B Using 1D CuO/TiO2 Nanofibers Synthesized via the Electrospinning Method
Source: Materials (Basel). 2025 Sep 11;18(18):4252. doi: 10.3390/ma18184252 (PMC12471999; doi:10.3390/ma18184252)
Supplement: Supplementary file 1 [file materials-18-04252-s001.zip › materials-3846388-supplementary.pdf]

Supplementary Material for

Photocatalytic degradation of Rhodamine B using 1D CuO/TiO_2 nanofibers synthesized via the Electrospinning Method

Shouzhen Duan [1], Wanjun Zhang [2], Xiaoyan Wang [1], Youqing Zhao [1], Hui Nan [1] and Guijun Yang [1,*]

[1] Qinghai University, Xining, 810016, China
[2] Huaghe Hydropower Development Co.Ltd. New Energy Branch, Xining, 810006, China
* Correspondence: junyygg@126.com

Academic Editor:

Received: 14 August 2025
Revised: 6 September 2025
Accepted: 8 September 2025
Published: date

Citation: Duan, S.; Zhang, W.; Wang, X.; Zhao, Y.; Nan, H; Yang, G. Photo-catalytic Degradation of Rhodamine B Using 1D CuO/TiO2 Nanofibers Synthesized Via the Electrospinning Method. Materials 2025, 18, x. https://doi.org/10.3390/xxxxx

Copyright: © 2025 by the authors. Submitted for possible open access publication under the terms and conditions of the Creative Commons Attribution (CC BY) license (https://creativecommons.org/licenses/by/4.0/).

Morphology Analysis

Furthermore, the CuO/TiO_2 samples were examined using EDS (Figure S1) in order to investigate the chemical structure of the materials. The results showed that the components of Ti, Cu and O were evenly distributed across the surfaces of the samples. The constituent distributions were consistent, which is strong evidence that the composite materials were correctly manufactured.

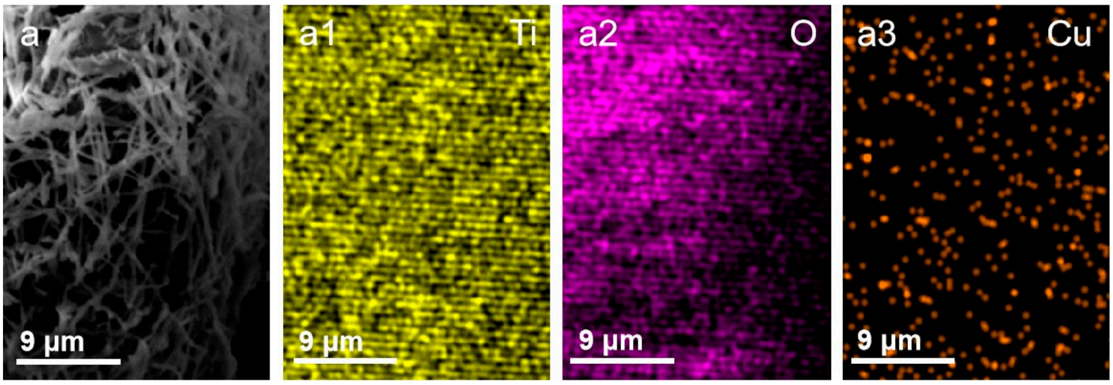

Figure S1. EDS image of CuO/TiO_2.

Catalytic performance

Table S1. Slope (K value) and coefficient of determination (R^2) in various fitted plots.

		CuO	TiO_2	CT-1	CT-2	CT-3	CT-4
Zero-level kinetic fit	$K/10^{-4}$	-5.32	-46.10	-81.90	-74.60	-47.30	-63.40
	R^2	89.31%	99.72%	95.59%	99.89%	98.88%	98.21%
First-level kinetic fit	$K/10^{-4}$	6.43	73.60	147.70	216.50	74.90	99.50
	R^2	89.20%	99.97%	89.70%	96.47%	97.97%	95.44%
Second-level kinetic fit	$K/10^{-4}$	119.20	7.76	284.50	713.70	120.20	160.70
	R^2	89.09%	99.80%	81.86%	86.95%	96.43%	91.33%

Table S2. Energy level data statistics.

Samples	Eg (eV)	CB (eV)	VB (eV)
TiO_2	3.05	-0.19	2.86
CuO	1.54	-1.49	0.05

Table S3. Efficiency comparison table of CuO/TiO_2 materials.

Samples	target object	degradation efficiency	Sources
$NiO/CuO/TiO_2$	methyl orange	120min 90%	[1]
CuO/TiO_2	tetracycline	60min 100%	[2]
$TiO_2/CuO/Chitosan$	methyl orange	240min 100%	[3]
$Ag/CuO/TiO_2$	rhodamine B	180min 82%	[4]
CuO/TiO_2	methyl orange	120min 73%	[5]
CuO/TiO_2	rhodamine B	75min 87%	This work

[1] Sivakumar S, A. Muthamilarasu, G. Divya, M. Sivakumar, et al. Ternary hybrid formation and Photocatalytic activity of $NiO/CuO/TiO_2$ microstructure over textile azo dye under solar light irradiation[J]. Research Square, 2021, http://dor.org/10.21203/rs.3.rs-912983/v1

[2] Sharma M, Mandal MK, Pandey S, et al. Visible-Light-Driven Photocatalytic Degradation of Tetracycline Using Heterostructured Cu_2O-TiO_2 Naotubes, Kinetics, Toxicity Evaluation of Degraded Products on Cell Lines[J]. ACS OMEGA, 2022, 7, 37, 33572-33586. https://pubs.acs.org/doi/10.1021/acsomega.2c04576

[3] Suhaila M, Azman Mr, Fatimah Z, et al. Solar-driven photodegradation of synthetic dyes by ternary of titanium oxide-copper oxide-chitosan catalyst[J]. Journal of Physics and Chemistry of Solids, 2023, 181, 111517. https://doi.org/10.1016/j.jpcs.2023.111517.

[4] Liu L, Xue Z, Sun Y, et al. Photocatalytic properties of TiO_2 nanofiber membranes co-modified with Ag and CuO[J]. Journal of Alloys and Compounds, 2025, 1038,182779. https://doi.org/10.1016/j.jallcom.2025.182779.

[5] Gao J, Mei X, Tang Y, Li D, Magnetron dual-target Co-sputtered CuO/TiO_2 heterojunction films for efficient photocatalytic applications[J]. Vacuum, 2025, 238, 114328. https://doi.org/10.1016/j.vacuum.2025.114328.
